# *FRMD3* gene: its role in diabetic kidney disease. A narrative review

**DOI:** 10.1186/s13098-015-0114-4

**Published:** 2015-12-30

**Authors:** Marjoriê Piuco Buffon, Denise Alves Sortica, Fernando Gerchman, Daisy Crispim, Luís Henrique Canani

**Affiliations:** Endocrine Division, Hospital de Clínicas de Porto Alegre, Federal University of Rio Grande do Sul, Rua Ramiro Barcelos 2350, prédio 12, 4° andar, Porto Alegre, RS 90035-003 Brazil; Endocrinology, Federal University of Rio Grande do Sul, Porto Alegre, RS Brazil; Federal University of Rio Grande do Sul, Porto Alegre, RS Brazil

## Abstract

Diabetic kidney disease (DKD) is a chronic complication of diabetes mellitus, which is considered a worldwide epidemic. Several studies have been developed in order to elucidate possible genetic factors involved in this disease. The *FRMD3* gene, a strong candidate selected from genome wide association studies (GWAS), encodes the structural protein 4.1O involved in maintaining cell shape and integrity. Some single nucleotide polymorphisms (SNPs) located in *FRMD3* have been associated with DKD in different ethnicities. However, despite these findings, the matter is still controversial. The aim of this narrative review is to summarize the evidence regarding the role of *FRMD3* in DKD.

## Background

Diabetes mellitus (DM) is a complex and chronic illness involving a state of hyperglycemia [1]. Depending on the intensity and duration of exposure to hyperglycemia, structural lesions may occur in the vascular endothelium and nervous tissue, causing damage and dysfunction in multiple organs and tissues, leading to chronic complications [2]. One such complication is diabetic kidney disease (DKD), in which there is increased urine excretion of proteins, predominantly albumin [3]. The precise mechanism that determines which patients with DM would or would not progress to renal failure and dialysis has yet to be elucidated. A great effort has been devoted to the study of candidate genes related to DKD development and progression.

## Diabetic kidney disease

DKD, which was initially described as a glomerular disease characterized by proteinuria, seems to be much more than that. Chronic kidney disease (CKD) is defined as renal damage resulting from structural or functional abnormalities of the kidneys, or glomerular filtration rate (GFR) <60 ml/min/1.73 m^2^ with or without kidney damage over a period of time equal to or greater than 3 months [4]. DKD affects around 25–30 % of individuals with DM and is responsible for over a quarter of new cases of end-stage renal disease (ESRD) [5, 6]. In 25 % of patients with DM, increased urinary albumin excretion (UAE) is observed [7]. Furthermore, low GFR has also been reported in a substantial proportion of normoalbuminuric subjects [7–10]. As a result, since 2006 the American Diabetes Association (ADA) has recommended DKD assessment based on UAE and estimated GFR (eGFR) [11, 12]. More recently, ADA has suggested that albuminuria must be classified as normal UAE or increased UAE [12], instead using the term microalbuminuria [13, 14].

Despite the high prevalence and severity of DKD, not all individuals with DM will develop this disease. The cumulative incidence of persistent albuminuria (microalbuminuria) in patients with type 1 diabetes mellitus (T1DM) has been reported from 12.6 % over 7.3 years according to The European Diabetes Prospective Complications Study Group (EURODIAB) [15] to 33 % in an 18-year study carried out in Denmark [16]. It seems that T1DM patients who do not develop DKD 10–15 years after diagnosis are protected from this complication [17]. Furthermore, some subjects with elevated albuminuria will progress to a more severe albumin profile and GFR loss, while others will remain stable or even return to normoalbuminuria [18, 19]. Thus, it is believed that the presence of risk factors such as hyperglycemia and hypertension will lead to DKD in genetically predisposed individuals.

## The genetics of diabetic kidney disease

Familial clustering of DKD, a multifactorial disease, has been reported in several studies, supporting the hypothesis that genetic factors are involved in its pathogenesis [20, 21]. However, all the efforts to identify a gene with a major effect have been disappointing, possibly because several genes are involved, acting synergistically or additively [22]. For instance, some genes might be related to increased albuminuria, and others to GFR decline [23, 24].

Pezzolesi et al. [25] conducted a genome wide association study (GWAS) in Caucasians with T1DM and found 13 SNPs (single nucleotide polymorphisms) associated with DKD. The most significant associations were identified in variants located near genes from four chromosomal regions: *CHN2/CPVL* on chromosome 7, *FRMD3* on chromosome 9, *CARS* on chromosome 11, and a locus near the *IRS2* on chromosome 13. Among those, the strongest association was near the *FRMD3* gene (*4.1 protein ezrin, radixin, moesin [FERM] domain containing 3*). Nevertheless, Maeda et al. [26] analyzed polymorphic variants of this gene [25] in Japanese with type 2 diabetes mellitus (T2DM) and did not find any association with DKD.

Regardless of extensive evidence of genetic susceptibility to DKD, the identification of susceptibility genes and their variants has had limited success [27, 28]. Mooyart et al. [29] conducted a meta-analysis of genetic association studies focusing on DKD and found that 21 genetic variants were significantly associated with DKD [29], two of them in the *FRMD3* gene. Other variants were in or near the following genes: *ACE, AKR1B1*, *APOC1, APOE, EPO, NOS3*, *HSPG2, VEGFA*, *CARS*, *UNC13B, CPVL, CHN2, GREM1*, and others. Additional variants were detected in subgroup analyses: *ELMO1* (Asians), *CCR5* (Asians), and *CNDP1* (T2DM). However, these associations were not confirmed by others [30].

The inconsistencies in the results of genetic association studies in complex diseases could be due to small sample sizes or incorrect associations conducting to false or spurious positive results [31–34]. Therefore, independent replication of positive associations remains essential to avoid associations by chance.

### *FRMD3* gene

**Fig. 1:**
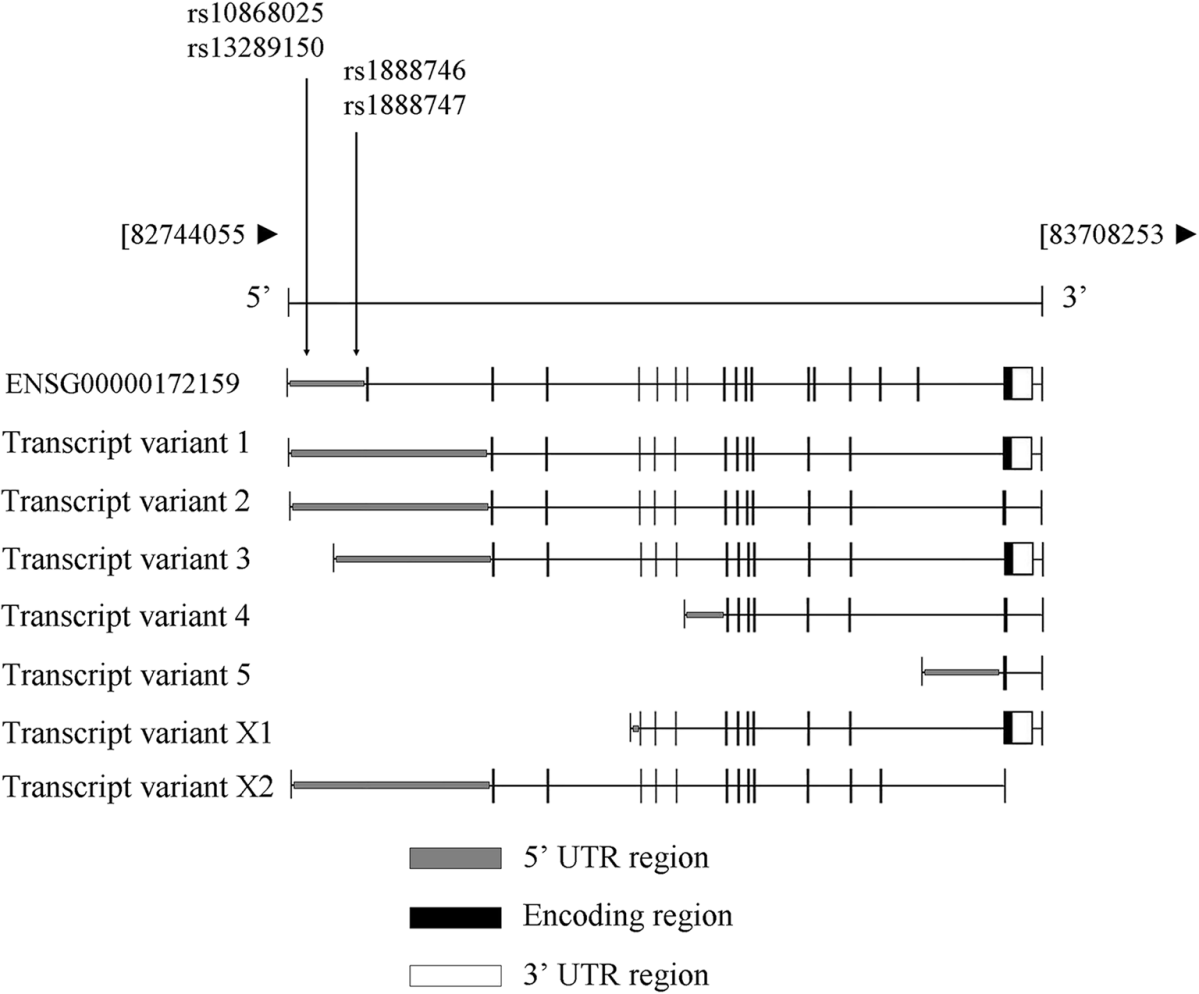
Map of the *FRMD3* gene in chromosome 9q21-32. The 21 exons (*boxes*) are shown from *left* to *right* according to the transcription region. *Black boxes* represent encoding regions, the *light grey box* represents the promoter—5′ UTR region, and the *white box* represents the 3′ UTR region. *Vertical arrows* show the polymorphic sites associated with diabetic kidney disease. Seven splice variants for the *FRMD3* gene are also shown in this figure. Figure adapted from http://www.ncbi.nlm.nih.gov/gene/257019 and http://www.ensembl.org

The *FRMD3* (ID: 257019) is located on chromosome 9, band q21.32. It has 21 exons spanning 2,282 base pairs (http://www.ncbi.nlm.nih.gov/gene/257019) (Fig. 1). In addition, 7 different mRNA sequence splice variants for the *FRMD3* gene are also shown in Fig. 1.

**Fig. 2:**
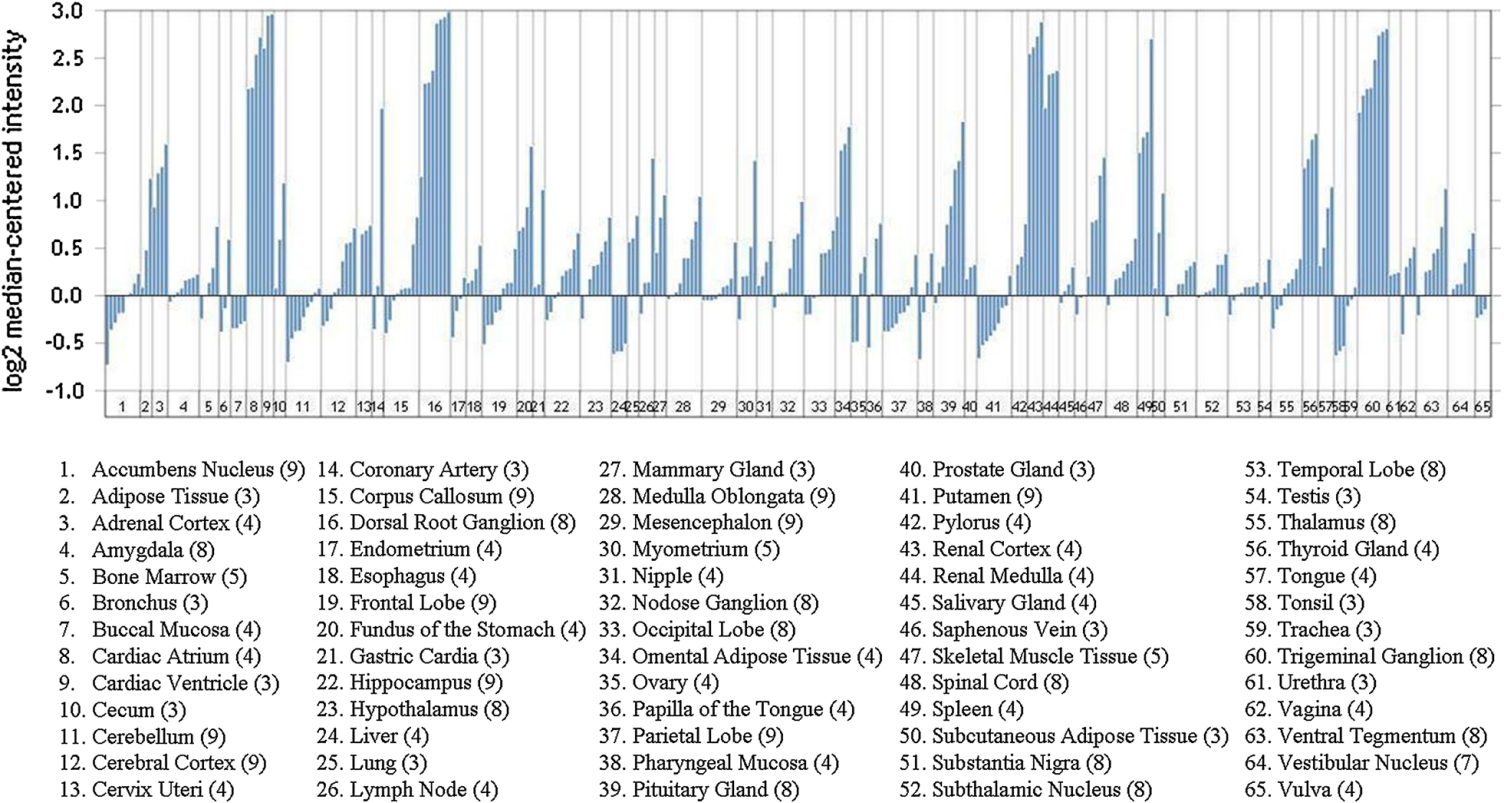
*FRMD3* expressions in a panel of different normal tissues. The graphic represents *FRMD3* expression in 65 normal tissues from 353 different samples. Data are expressed as fold change, and the number shown after each tissue represents the sample size analyzed for this respective tissue. Data were extracted from the Nephromine database (Life Technologies, Ann Arbor, MI; http://www.nephromine.org)

**Fig. 3:**
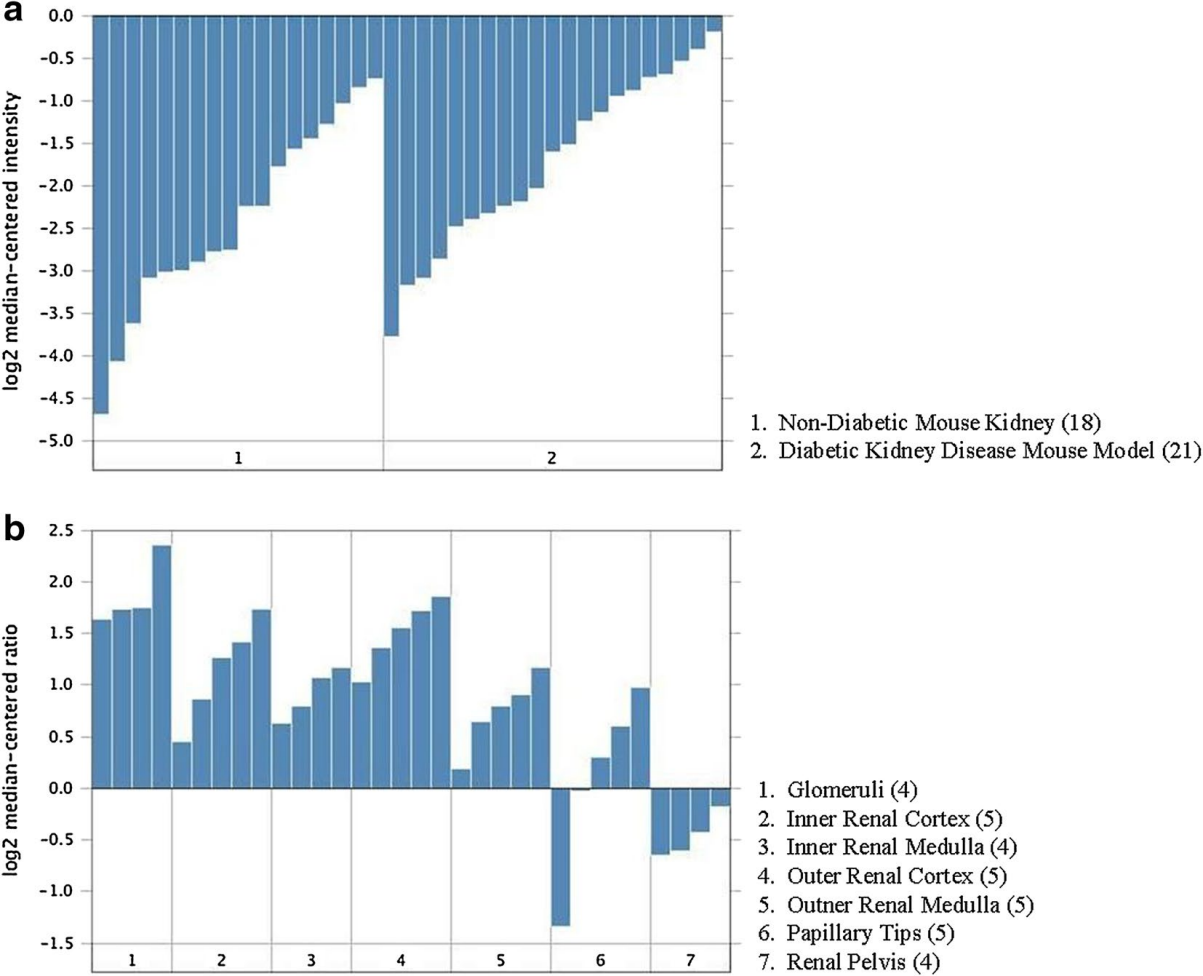
*FRMD3* expression in kidney. **a** *FRMD3* expression in Hodgins mice according to the presence of diabetic kidney disease. The graphic represents mRNA expression from 39 kidney samples. **b** *FRMD3* expression in a normal kidney from Higgins mice according to different kidney regions. The graphic represents mRNA expressions from 34 kidney samples. Data are expressed as fold change, and were extracted from the Nephromine database (Life Technologies, Ann Arbor, MI; http://www.nephromine.org)

*FRMD3* encodes protein 4.1O, a structural protein that is part of the protein 4.1 family [35]. Members of this family act as cytoskeletal proteins, maintaining cell shape and integrity in a variety of cell types including rat nephron cells [36, 37]. However, the role of protein 4.1O has not yet been elucidated [38]. It contains a FERM domain, which contributes to cell integrity by interacting with transmembrane proteins and actin filaments [38, 39]. *FRMD3* is detected in different tissues according to the Nephromine database (Fig. 2). Studies also have reported *FRMD3* expression in adult ovaries, fetal skeletal muscle, brain, thymus, and human podocytes [35]. In addition, *FRMD3* expression seems to be decreased in a DKD mouse model as compared to non-diabetic mouse kidneys (Fig. 3a). *FRMD3* is differentially expressed in different regions of murine kidney, showing the highest level in glomeruli (Fig. 3b).

**Fig. 4:**
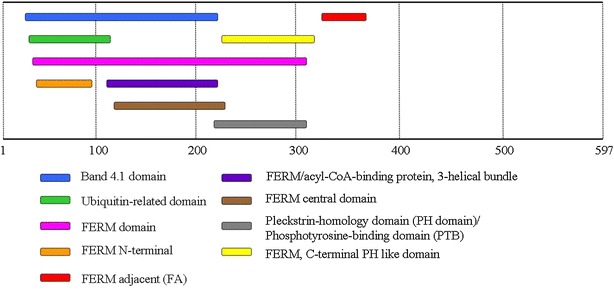
Different domains of the FRMD3 protein. The various domains are depicted according to the specific length of each domain. The complete protein length is 597 amino acids. Figure adapted from http://www.ebi.ac.uk/interpro/protein/A2A2Y4

The 4.1 protein family comprises a group of proteins including 4.1R, 4.1G (general type), 4.1B (brain type), and 4.1N (neuron type) [40]. Erythrocyte protein 4.1 (4.1R) is a multifunctional protein essential for maintaining erythrocyte shape, mechanical properties of the membrane, deformability, and stability through interactions with the spectrin and actin skeleton network [40]. Figure 4 represents different domains of the FRMD3 protein. The FERM domain [38] is an N-terminal 30-kDa domain containing binding sites for cytoplasmatic tails of integral membrane proteins such as band 3 (integral membrane protein involved in spectrin-actin interaction) [41, 42], glycophorin C (erythroid membrane proteins) [43], CD44 (transmembrane glycoprotein found in erythroid as well as non-erythroid cells) [44], p55 (abundantly palmitoylated phosphoprotein of the erythroid membrane) [43] and calmodulin (a highly conserved calcium-binding protein, ubiquitously distributed in eukaryotic cells, known to affect a plethora of biological functions) [40]. An internal 8-10 kDa domain contains the spectrin-actin binding activity that is necessary for membrane stability [45], and the 22–24 kDa C-terminal domain has been reported to bind immunophilin FKBP13 (membrane-associated protein thought to function as an endoplasmic reticulum chaperone) [46] and nuclear mitotic apparatus protein (NuMA) [47].

FERM domain proteins have been implicated in nephron function. Some of these proteins were described as important in the composition of specialized cell–cell adhesion complexes. These complexes called slit-diaphragms that form between podocyte foot processes surrounding glomerular blood vessels have an important role in the function of podocytes, establishing the blood filtration barrier in the kidney glomerulus. A reduced expression of some FERM domain proteins in zebrafish, such as nephrin, podocin or band 4.1/FERM domain mosaic eyes resulted in loss of glomerular filtration discrimination and abnormal passage of high molecular weight substances into the glomerular filtrate [48]. Another FERM domain protein that has been recently related to nephron function is kindlin, an adaptor protein that contributes to renal tubulointerstitial fibrosis. This protein regulates renal tubular cell plasticity by activation of Ras and its downstream signaling pathway [49]. Therefore, it is possible that *FRMD3* might have related functions in kidney.

**Fig. 5:**
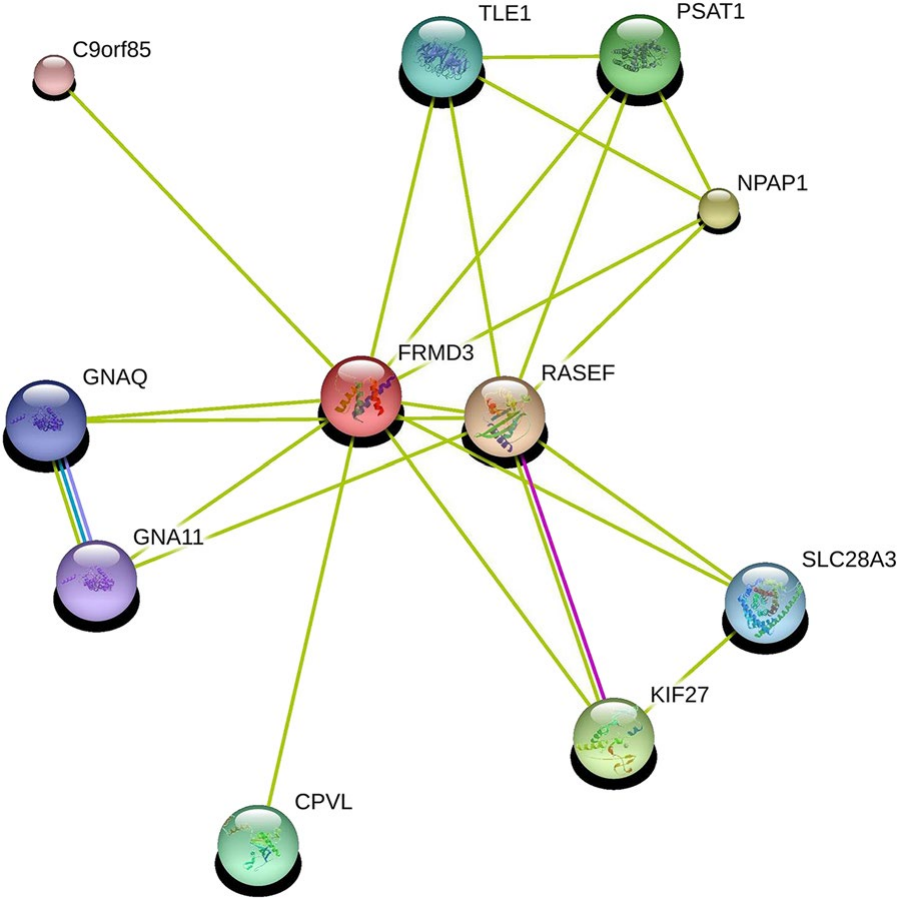
FRMD3 protein interaction view networks in humans. The figure represents predicted interactions of FRMD3 with different proteins using medium confidence algorithms. *Lines of different colors* indicate that the respective interaction was predicted using more than one algorithm/tool. *RASEF* RAS and EF-hand domain containing, *NPAP1* nuclear pore associated protein 1, *KIF27* kinesin family member 27, *PSAT1* phosphoserine aminotransferase 1, *CPVL* carboxypeptidase, vitellogenic-like, *TLE1* transducin-like enhancer of split 1 (E(sp1) homolog, Drosophila), *SLC28A3* solute carrier family 28 (sodium-coupled nucleoside transporter), member 3, *GNAQ* guanine nucleotide binding protein (G protein), q polypeptide, *GNA11* guanine nucleotide binding protein (G protein), alpha 11 (Gq class), *C9orf85* chromosome 9 open reading frame 85. Figure extracted from database Search Tool for the Retrieval of Interacting Genes/Proteins STRING.db (http://string-db.org/)

In the study of Pezzolesi et al. [25], *FRMD3* expression was increased in proximal renal tubular human cells. Expression data for *FRMD3* and its co-expressed transcripts suggest that these genes are linked to early development of DKD [50]. Moreover, *FRMD3* is predicted to interact with other genes, suggesting alternative pathways in which this gene might act (Fig. 5).

It has been hypothesized that the 9q21.32 locus contributes to glomerular injury early in the pathogenesis of DKD [51]. More recently, the rs1888747 risk allele was shown to generate a transcription factor binding site (TFBS) in a module that is shared by multiple members of the bone morphogenetic protein (BMP) signaling pathway, which has previously been implicated in the development of DKD [50, 52]. Regarding these findings, new pathways are being proposed to explain the role of *FRMD3* in DKD physiopathology.

### Variants in the *FRMD3* gene and diabetic kidney disease

Research has been developed to investigate the association of variants in the *FRMD3* gene and risk of DKD. The main variants studied so far are briefly described in Table 1. The polymorphisms rs942278, rs942280, rs942283, rs1535752, rs1535753, rs2378658, rs10867977 and rs10867977 are in strong linkage disequilibrium (LD) with each other. The Hapmap project does not show LD information for the rs1888746, rs1888747, rs10868025 and rs13289150 SNPs (http://hapmap.ncbi.nlm.nih.gov).

**Table 1:** *FRMD3* variants studied in the context of diabetic kidney disease

Variant	Reference	Ethnicity	Sample description	Chromossome 9^a^ position	Risk allele	P value	Odds ratio (95 % CI)
rs942278	Freedman et al. [55]	African–American	966 cases (T2DM-DKD) 1032 controls (Non-DM, non DKD)	85906066	T	0.0023	1.30 (1.11–1.53)
rs942280				85905861	G	0.00070	1.28 (1.09–1.51)
rs942283				85905666	C	0.0014	1.25 (1.07–1.47)
rs1535752				85905253	T	0.013	1.27 (1.03–1.57)
rs1535753				85905060	T	0.0024	1.24 (1.06–1.46)
rs2378658				85904778	C	0.0040	1.25 (1.06–1.46)
rs10867977				85907516	G	0.022	1.31 (1.06–1.62)
rs1888746	Hu et al. [53]	Chinese	622 cases (378 T2DM-DKD and 244 DKD and retinopathy) 280 controls (>10 years T2DM)	86155392	C	0.66	0.93 (0.60–1.38)
	Pezzolesi et al. [25]	Caucasian	820 cases (284—proteinuria and 536—ESRD) 885 controls (T1DM)	86155392	C	0.02	NA^b^
rs1888747	Maeda et al. [26]	Japanese	754 cases (T2DM- DKD and retinopathy) and 558 controls (T2DM—only retinopathy)	86155551	G	0.10	1.24 (0.96–1.61)
			449 cases (T2DM—DKD and retinopathy) and 965 controls (T2DM- only retinopathy)			0.84	1.02 (0.83–1.26)
			32 cases (T2DM-microalbuminuria progressed to overt proteinuria) and 168 controls (T2DM-microalbuminuria)			0.004	0.28 (0.12–0.67)
			300 cases (T2DM-DKD) and 224 controls (normoalbuminuria and >10 years T2DM)			0.43	0.87 (0.61–1.23)
	Pezzolesi et al. [25]	Caucasian				0.00000067	1.45 (1.25–1.67)
	Williams et al. [30]	Caucasian	UK—ROI: 903 cases (DKD, retinopathy and ESRD) and 1001 controls (T1DM) FinnDiane: 1289 cases (ESRD and macroalbuminuria) and 1577 controls (normoalbuminuria) Reanalysis of US GoKind data: 905 cases (DKD) and 898 controls (T1DM)			0.24	1.06 (0.96–1.17)
	Pezzolesi et al. [58]	Caucasian	743 cases (T2DM- 427 microalbuminuria and 316 proteinuria/ESRD) and 646 controls (T2DM-normoalbuminuria)			0.92	1.01 (0.86–1.19)
	Pezzolesi et al. [51]	Caucasian	382 controls (non T2DM) and 416 cases (T2DM- DKD)			0.0014	NA^d^
	Moyaart et al. [29]	Caucasian	Meta-analysis 1052 cases (DKD) 2057 controls (T1DM)			0.60	0.74 (0.65–0.83)
rs10868025	Pezzolesi et al. [25]	Caucasian		86164176	A	0.00005	1.45 (1.25–1.67)
	Williams et al. [30]	Caucasian				0.19	1.06 (0.97–1.17)
	Pezzolesi et al. [51]	Caucasian				0.0039	NA^e^
	Pezzolesi et al. [58]	Caucasian				0.90	0.99 (0.84–1.16)
	Maeda et al. [26]	Japanese	754 cases (T2DM-DKD and retinopathy) and 558 controls (T2DM- only retinopathy)			0.31	1.12 (0.89–1.42)
			449 cases (T2DM-DKD and retinopathy) and 965 controls (T2DM- only retinopathy)			0.54	0.94 (0.78–1.14)
			32 cases (T2DM-microalbuminuria progressed to overt proteinuria) and 168 controls (T2DM-microalbuminuria)			0.07	0.49 (0.22–1.06)
			300 cases (T2DM-DKD) and 224 controls (normoalbuminuria and >10 years T2DM)			0.33	0.85 (0.62–1.18)
	Hu et al. [53]	Chinese				0.25	0.91 (0.76–1.07)
	Moyaart et al. [29]	Caucasian				0.12	0.72 (0.64–0.81)
rs13289150	Pezzolesi et al. [25]	Caucasian		83549513	A	0.05	NA^c^

*NA* not applicable, *T2DM* type 2 diabetes mellitus, *T1DM* type 1 diabetes mellitus, *DKD* diabetes kidney disease, *ESRD* end-stage renal disease

^a^Adapted from on the hg19 genome assembly

^b^Hazard ratio 1.33

^c^Hazard ratio 1.23

^d^Z score 3.19

^e^Z score 2.88

The first published study regarding variants in *FRMD3* was a GWAS with T1DM patients from the GoKinD (Genetics of Kidneys in Diabetes) collection [25]. The SNP rs10868025 had the strongest association with DKD (OR = 1.45, P = 5.0 × 10^−7^) [25]. This SNP involves a G for A nucleotide substitution and is located in the long arm of chromosome 9 at position 85.4, near the 5′ end of *FRMD3* [25]. The frequency of the risk allele was 0.66/0.56 (cases/controls) in the Joslin Diabetes Center study (JDC study) and 0.66/0.59 in the George Washington University study (GWU study) [25]. Two other *FRMD3* SNPs associated with DKD in the GoKinD collection (rs1888746 and rs13289150) were also associated with the development of severe nephropathy in the DCCT/EDIC (Diabetes Control and Complications Trial/Epidemiology of Diabetes Interventions and Complications) follow-up study [25].

In a meta-analysis, involving three studies including 1052 cases and 2057 controls, the rs10868025 SNP was associated with a lower risk of DKD in European T1DM subjects (OR 0.72 [95 % CI 0.64–0.81]) [29], whereas in a study with T2DM subjects [26], no significant associations between the rs10868025 SNP and DKD were detected in four independent Japanese populations. The G allele frequency for cases vs. controls in the four populations studied was: 0.74 vs. 0.73, 0.72 vs. 0.73, 0.67 vs. 0.73, and 0.70 vs. 0.73 (P > 0.25 in all cases). The same findings were replicated in the Chinese population [53], for which no association between *FRMD3* SNPs and DKD was observed (P = 0.249).

The rs1888747 (C/G) SNP is intergenic, located on the long arm of chromosome 9 at position 85.3, near the promoter region of the gene [54]. In the study developed by Pezzolesi et al. [25], a relevant association of rs1888747 SNP was found with DKD (P = 6.3 × 10^−7^) in Caucasians with T1DM. The frequency of the risk allele (G) was 0.68/0.73 for controls/cases in the GWU GoKinD and 0.66/0.74 for controls/cases in the JDC GoKinD [25]. These findings were strengthened by a meta-analysis including three studies analyzing the rs1888747 SNP in a total of 1052 cases and 2057 controls [29], which confirmed an association of rs1888747 SNP with DKD (OR 0.74 [95 % CI 0.65–0.83]; P = 0.602) [29], primarily driven by the GoKinD cohort. However, in Japanese individuals with T2DM, no associations between rs1888747 SNP and DKD were demonstrated in four different groups [26]. The frequency of the G allele for cases/controls was 0.81/0.80, 0.80/0.80, 0.70/0.82 and 0.77/0.81 (P > 0.05).

In 2011, Freedman et al. [55] identified that a potential interaction between *FRMD3* and the *APOL1* (apolipoprotein L1 gene)—*MYH9* (non-muscle myosin heavy chain 9 gene) region on chromosome 22 contributes to DKD susceptibility in African Americans. Nevertheless, initially no association was found between *FRMD3* SNPs (rs942280 and others, Table 1) and DKD before adjusting for variants on chromosome 20. The results revealed an approximately 25–30 % increase in DKD risk with multiple *FRMD3* SNPs in subjects not homozygous for *MYH9* risk haplotypes (or *APOL1*risk variants).

One year later, Williams et al. [30] formed the GEnetics of Nephropathy, an International Effort (GENIE) consortium, to examine reported genetic associations with DKD in T1DM previously observed in the GoKinD collection [25]. The samples were provided by the All Ireland-Warren 3-Genetics of Kidneys in Diabetes UK and Republic of Ireland (UK–ROI) collection and the Finnish DKD Study (FinnDiane). None of the analyzed *FRMD3* polymorphisms were associated with DKD (rs1888747 [P = 0.77 and 0.25, UK–ROI and FinnDiane respectively]; rs1086805 [P = 0.52 for UK–ROI and 0.25 for FinnDiane]) [30].

Further evidence of the role of *FRMD3* in kidney disease came from the study of Park et al. [56]. In this study, the authors aimed to find candidate genetic determinants of renal function in 1007 individuals from 73 extended families of Mongolian origin [56]. The strongest associations found were with rs17400257 SNP (P = 7.21 × 10^−9^) and rs6559725 SNP (P = 9.12 × 10^−7^). The rs17400257 SNP is located 45 kb downstream of *FRMD3*, and rs6559725 SNP is located in the intronic region of *FRMD3* [56].

After the initial findings in T1DM in 2009 [25], Pezzolesi et al. [51] proceeded to examine whether the SNPs at these susceptibility loci were associated with DKD in a T2DM Family Collection. The 9q21.32 locus was significantly associated with high microalbuminuria, proteinuria, and ESRD [51]. Among diabetic family members, rs1888747 SNP on chromosome 9q21.32 was associated with advanced nephropathy (P = 0.029). Furthermore, when the definition of DKD was expanded to include individuals with high microalbuminuria, the strength of the association improved significantly [51].

Martini et al. [50] proposed a transcriptional link that might explain how the rs1888747 SNP in *FRMD3* influences transcriptional regulation within the bone morphogenetic protein (BMP)-signaling pathway. *FRMD3* transcript levels decreased significantly with the progression of DKD (P < 0.02). *FRMD3* gene expression was studied by comparing renal biopsies in a group of 22 Pima Indians with T2DM and normal GFR and a cohort of seven participants with T2DM and CKD stage 3 [50]. Hierarchical clustering using the *FRMD3* coexpressed transcripts detected two distinct clusters. In cluster 1, presenting higher ∆ACR/year (∆ albumin-to-creatinine ratio per year), gene expressions of seven out of the eight BMP pathway genes (*BMPR2*, *CREB1*, *KRAS*, *MAP3K7*, *PRKAR2B*, *SMAD5*, and *XIAP*) were lower than the expressions of these genes in cluster 2. These findings suggest a common molecular mechanism responsible for the coregulation of *FRMD3* and several BMP pathway members [50]. Additionally, in silico comparison of sequence variants of the risk allele identified a potential homeodomain factor (HOMF) transcription factor binding site (TFBS) covering the SNP position [50]. The rs1888747 SNP affects protein binding, suggesting the generation of a TFBS by this particular SNP [50]. However, the mechanism mediating the connection between *FRMD3* and BMP pathway members is still unknown, and no data are available at protein, RNA, or microRNA levels to elucidate this association [50]. It should be noted that Palmer and Freedman [54] suggested that the framework proposed by Martini et al. [50] is limited to genes with known or predicted functional roles.

Recently, a GWAS was performed by Palmer et al. [57] with the objective of evaluating candidate DKD susceptibility genes in African Americans. The SNPs selected were the same previously studied in European ancestries by Pezzolesi et al. [25]. The results showed that SNPs in *FRMD3* tended toward association with T2DM-ESRD (P < 0.05).

## Conclusions

Even though not much is known about the *FRMD3* gene, some studies provide insights into the relationship between *FRMD3* variants and DKD. Major findings in the African American population show an association between DKD and many SNPs, especially rs10867977 (OR 1.31, 95 % CI 1.06–1.62). In the Japanese population, a significant association was found only at rs1888747, whereas in Caucasians three SNPs have been found to be associated with DKD (rs1888746, rs1888747, rs10868025). Nevertheless, considering all SNPs and ethnic groups analyzed, the strongest association was found in Caucasians with polymorphisms rs1888747 and rs10868025. However, the data produced so far is not sufficient to ascertain a role of *FRMD3* in DKD pathogenesis. Also, because the relationship between the *FRMD3* gene and DKD was recently detected, very few studies focusing on its mRNA and protein expression are available. Some limitations regarding the development of genetic association studies for diabetic complications could explain differences among studies which evaluated the *FRMD3* gene. The first limitation involves differences in the definition of DKD among the studies. The recommended prognosis of DKD is determined by two parameters, GFR and albuminuria levels. Nevertheless, the majority of studies frequently use only one of these parameters to classify the disease. Moreover, differences in confounding factors such as DM duration and glycemic control could also influence the results.

The functional context of *FRMD3* could be experimentally elucidated by several approaches. One possibility is to perform functional studies in kidney cells to evaluate the consequences of *FRMD3* gene silencing/overexpression. Knockout/transgenic mouse models may also contribute to clarify FRMD3 function in the kidney. Fine mapping studies of GWAS data could identify new functional FRMD3 polymorphisms associated with DKD. Moreover, designing studies would be helpful to determine what molecules could be interacting with *FRMD3* aiming to further increase knowledge of the mechanisms and pathways in which the gene is involved and its relation to diseases, as suggested by Martini et al. [50].

In summary, *FRMD3* is a strong candidate gene for DKD. However, further studies are needed to explain the pathways through which *FRMD3* influences the onset of this diabetic complication.

## Abbreviations

DKD: diabetic kidney disease
GWAS: genome wide association studies
SNPs: single nucleotide polymorphisms
DM: diabetes mellitus
CKD: chronic kidney disease
ESRD: end-stage renal disease
UAE: urinary albumin excretion
ADA: American diabetes association
eGFR: estimated GFR
T1DM: type 1 diabetes
EURODIAB: the European diabetes prospective complications study
FRMD3: 4.1 protein ezrin, radixin, moesin [FERM] domain containing 3
T2DM: type 2 diabetes mellitus
TFBS: transcription factor binding site
BMP: bone morphogenetic protein
GoKinD: genetics of kidneys in diabetes
JDC: joslin diabetes center
GWU: George Washington university
DCCT/EDIC: diabetes control and complications trial/epidemiology of diabetes interventions and complications
APOL1: apolipoprotein L1
MYH9: non-muscle myosin heavy chain 9
GENIE: Genetics of nephropathy—an international effort
UK–ROI: Republic of Ireland
FinnDiane: finnish DKD study
HOMF: homeodomain factor
